# BING, a novel antimicrobial peptide isolated from Japanese medaka plasma, targets bacterial envelope stress response by suppressing *cpxR* expression

**DOI:** 10.1038/s41598-021-91765-4

**Published:** 2021-06-09

**Authors:** Miao Dong, Shu Hin Kwok, Joseph L. Humble, Yimin Liang, Sze Wing Tang, Kin Hung Tang, Man Kit Tse, Josh Haipeng Lei, Rajkumar Ramalingam, Mohamad Koohi-Moghadam, Doris Wai Ting Au, Hongyan Sun, Yun Wah Lam

**Affiliations:** 1 Department of Chemistry, City University of Hong Kong, Kowloon, Hong Kong SAR China; 2 Present Address: Institute of Biodiversity, Animal Health and Comparative Medicine, University of Glasgow, Glasgow, UK; 3 Present Address: Faculty of Health Sciences, University of Macau, Taipa, Macau SAR China; 4 Present Address: Applied Oral Sciences and Community Dental Care, University of Hong Kong, Pokfulam, Hong Kong SAR China

**Keywords:** Drug discovery, Microbiology

## Abstract

Antimicrobial peptides (AMPs) have emerged as a promising alternative to small molecule antibiotics. Although AMPs have previously been isolated in many organisms, efforts on the systematic identification of AMPs in fish have been lagging. Here, we collected peptides from the plasma of medaka (*Oryzias latipes*) fish. By using mass spectrometry, 6399 unique sequences were identified from the isolated peptides, among which 430 peptides were bioinformatically predicted to be potential AMPs. One of them, a thermostable 13-residue peptide named BING, shows a broad-spectrum toxicity against pathogenic bacteria including drug-resistant strains, at concentrations that presented relatively low toxicity to mammalian cell lines and medaka. Proteomic analysis indicated that BING treatment induced a deregulation of periplasmic peptidyl-prolyl isomerases in gram-negative bacteria. We observed that BING reduced the RNA level of *cpxR*, an upstream regulator of envelope stress responses. *cpxR* is known to play a crucial role in the development of antimicrobial resistance, including the regulation of genes involved in drug efflux. BING downregulated the expression of efflux pump components *mexB*, *mexY* and *oprM* in *P. aeruginosa* and significantly synergised the toxicity of antibiotics towards these bacteria. In addition, exposure to sublethal doses of BING delayed the development of antibiotic resistance. To our knowledge, BING is the first AMP shown to suppress *cpxR* expression in Gram-negative bacteria. This discovery highlights the *cpxR* pathway as a potential antimicrobial target.

## Introduction

The emergence of antimicrobial resistance (AMR) in microorganisms is one of the major global threats to human health. There is a dire need for new antibacterial drugs, but the number of new FDA approved antibacterial drugs has significantly fallen since the late twentieth century, from 29 in the 1980s to only 9 in the 2010s^1^. A recently published “roadmap for antibiotic development”^7^ recommended the identification and optimization of natural products with antibacterial activities, especially against Gram-negative organisms. It also calls for more research on combinational therapies by exploring the potential synergistic effects of antibacterial agents.

One of the most promising classes of natural antibacterial agents is the antimicrobial peptide (AMP). To date, more than 3000 experimentally validated AMPs have been reported, among which 70% show antibacterial activities^2^. AMPs are short (4–50 amino acid residues) peptides that belong to a variety of structural classes^3,4^. Around 80% of all reported AMPs have an overall cationic charge, but with a relatively high (30–80%) proportion of hydrophobic residues^5,6^. It is generally believed that this combination of biochemical properties allows peptides to penetrate bacterial surfaces by attaching to or inserting into the membrane bilayers. Some AMPs are known to demonstrate other cytotoxic mechanisms such as the suppression of cell wall biosynthesis and blockage of the synthesis of nucleic acids and proteins^4,7^. Recent findings have indicated that antibiotic-resistant bacteria are generally collaterally sensitive to a wide range of AMPs^8^ and that AMPs are less likely to induce resistance^9,10^. These characteristics make AMPs a promising candidate in combinatory anti-bacterial therapies^11^.

The past two decades have seen the identification of more than 100 new AMPs every year^12^, from a long list of organisms^13–16^. However, three aspects of AMP research have been relatively unexplored. First, existing AMPs have been identified from a narrow range of animal secretions and tissues, such as mucus^17,18^, liver^19^ and kidney^20^. Blood is one of the foremost immunological tissues, which contains many antibacterial proteins. Although AMPs have been identified in circulating cells^21,22^, blood plasma has rarely been used as a resource for AMP discovery: only five AMPs have been identified in plasma so far^23,24^. Secondly, although the number of AMPs has been growing steadily, most of them were discovered in terrestrial organisms. AMPs from aquatic organisms are underrepresented^25^. Of all the published AMPs to date, only 136 were identified in fish, most of them through their sequence homology with known AMPs previously reported from other species. As marine-derived AMPs often exhibit different structural properties from their territorial counterparts^25^, this approach may limit the discovery of AMPs with novel antibacterial mechanisms. Finally, while the general antibacterial mechanisms of cationic amphipathic peptides have been extensively described^3^, some AMPs have been shown to bind to non-canonical molecular targets^26,27^, suggesting that the range of antibacterial mechanisms utilized by AMPs is not fully understood. Due to the lack of mechanistic understanding of AMP functions, the rational design of combined therapies, either with other AMPs^28^ or small-molecule antibiotics^29^, is still not comprehensive.

To overcome these research gaps, we explored, for the first time, fish blood as a source of AMPs. To achieve this goal, we use a systematic proteomic approach to build a database of circulating peptides in Japanese medaka (*Oryzias latipes*). High throughput proteomics has been applied to characterize the plasma proteins in small fish models such as zebrafish and medaka^30,31,32^. The identified peptides are then filtered through a bioinformatics pipeline, resulting in a shortlist of potential AMPs, which are experimentally validated against gram positive and negative bacteria. This led to the discovery of a novel AMP with a previously unknown bactericidal mechanism.

## Results and discussion

### Characterisation of short circulating peptides in *O. laptipes*

We aimed to build a database of short peptides in the plasma of Japanese medaka and thereby increase our understanding of circulating AMPs in fish. This was achieved by subjecting short peptides isolated from medaka plasma to mass spectrometric analysis. We envisaged that the composition of the circulating peptides is highly dynamic, and dependent on conditions such as sex, age and health status. To avoid biases due to the predominant expression of certain types of peptides associated with specific conditions, we pooled the plasma collected from fishes of different sexes and ages. We also included bacterially infected fish in the super-mix to allow for the detection of potential infection-associated peptides. Since the primary aim of this study is to discover novel AMPs with therapeutic potentials, we focused our characterisation on peptides with the molecular weight under 3 kDa (shorter than 30 amino acids), which are better candidates for clinical translation.

**Figure 1:**
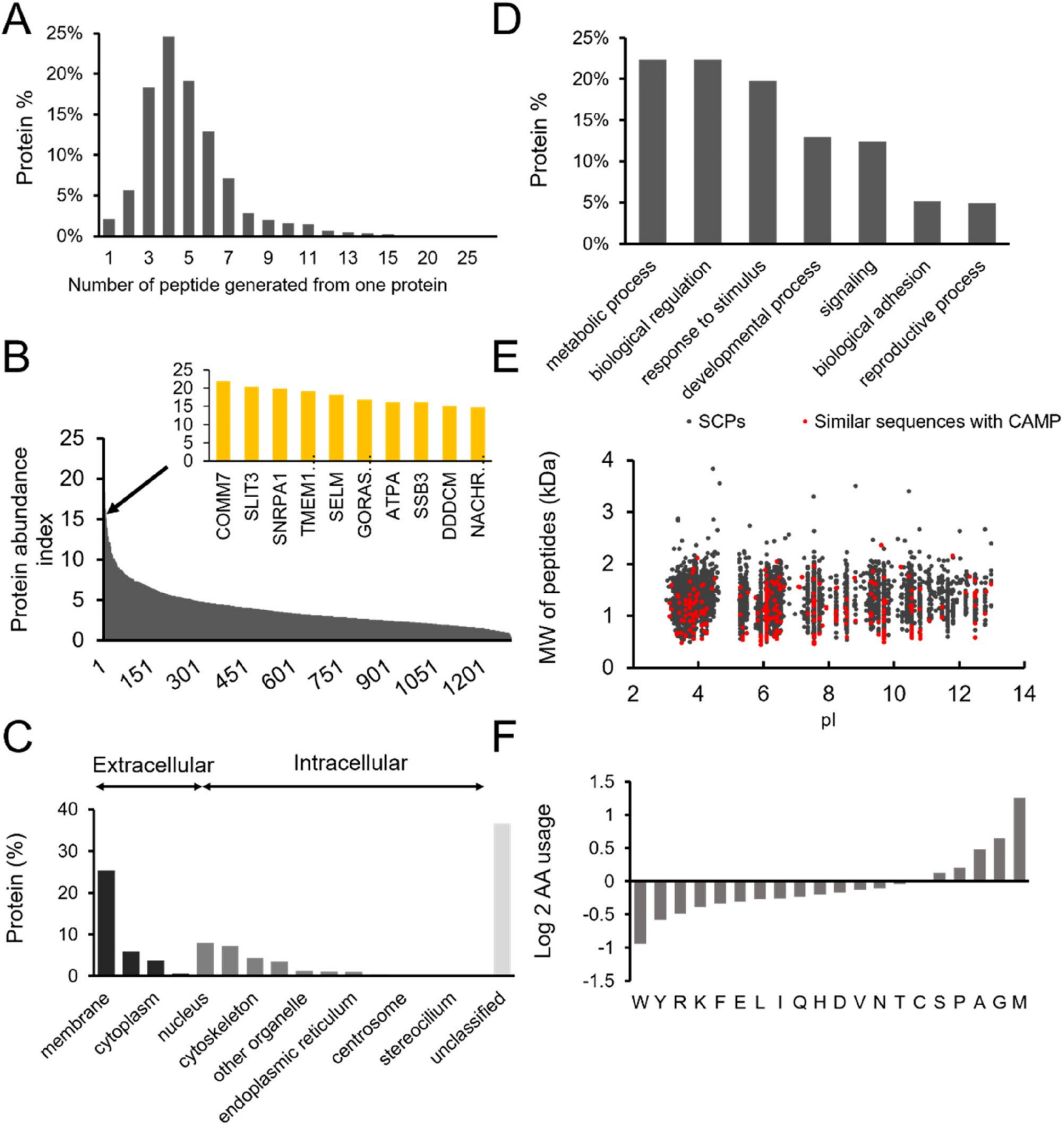
Characteristics of fish SCPs. (**A**) Distribution of SCP numbers produced per parent protein. (**B**) Identity of proteins that give rise to the highest number of SCPs. (**C**,**D**) Gene ontology enrichment analysis of parent proteins in terms of biological processes and cellular components. (**E**) Distribution of molecular weight and isoelectric points of all SCPs identified in this study. Red dots represent SCPs that are homologous to existing AMPs. (**F**) Amino acid usage of SCPs, relative to amino acid usage of available medaka proteome.

Using this approach, we identified 6399 unique peptide sequences. As far as we know, this is the largest database of short circulating peptides (SCPs) to date and will be an invaluable resource for better understanding the generation and functions of peptides in animal blood. The detected SCPs were mapped to 1289 proteins. More than 60% of the parent proteins produce 3–5 peptides, with a small number of proteins giving rise to over 20 peptides (Fig. 1A). Interestingly, the parent proteins that generated the highest number of SCPs, such as COMM domain-containing protein 7-like (COMM7) and uncharacterized protein SLIT3^31,33^, are not among the most abundant proteins detected in medaka serum (Fig. 1B). This suggests that SCPs may not be a result of the nonspecific degradation of serum proteins. In addition, 23% of these parent proteins are intracellular (Fig. 1C,D). It is possible some of these SCPs were selectively secreted and/or retained in the plasma from their tissue origins. We analysed the physiochemical properties of the detected SCPs. As expected, more than 99% of the SCPs are less than 3 kDa. There was a slight bias towards acidic peptides within the SCPs (Fig. 1E). The amino acid usage of the SCPs, when compared to that of the general medaka proteome, revealed a preference towards nonpolar amino acids such as methionine, glycine and alanine, while aromatic amino acids are underrepresented (Fig. 1F). Future research will be focused on how these properties affect the maintenance of peptides in circulation.

### Prediction of novel antimicrobial peptides

A comparison of the sequences between our collection of SCPs and the 2767 experimentally validated AMPs in the CAMP database (CAMP_R3_)^34^ revealed that 301 of the SCPs shared significantly sequence homology with previously reported AMPs. Of these 301 peptides, only 17 have previously been reported in other fish species. Up to 20% of these AMPs have been reported in amphibian skin secretions, including the Rugosin, Ascaphin and Gaegurin families^34^. Interestingly, Cystatin, the largest known family of AMPs, is not represented in the SCPs. This suggests that the distribution of AMP types in fish might be different from that in terrestrial organisms.

**Figure 2:**
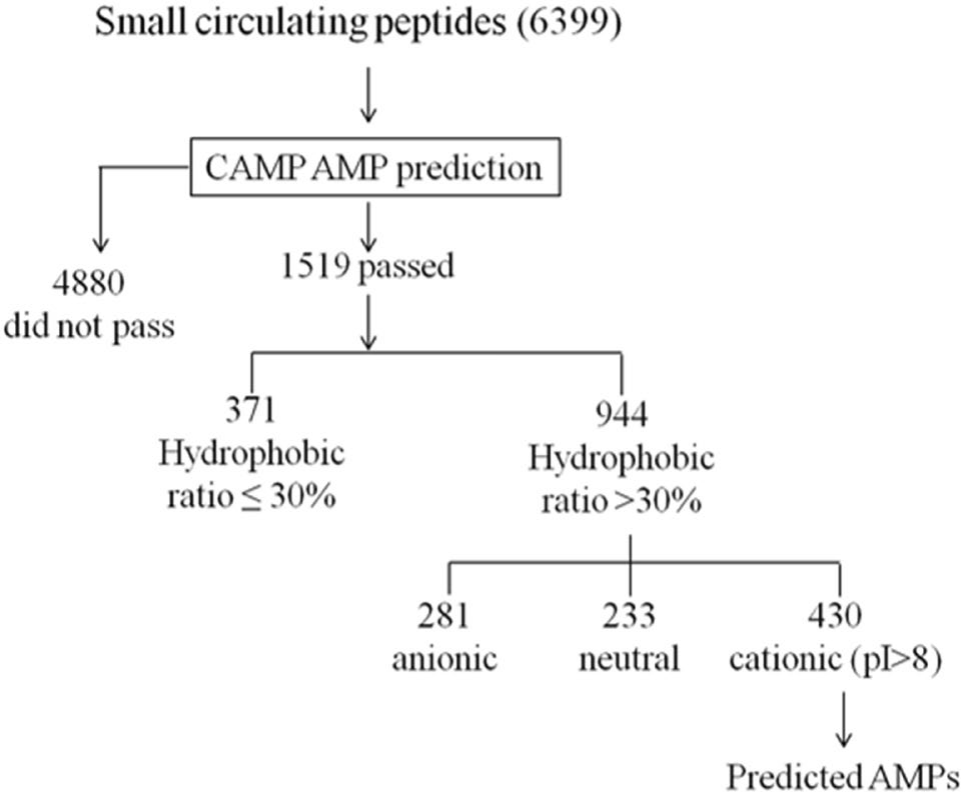
The AMP prediction pipeline of this study.

Of the remaining SCPs, 1519 peptides (24%) were predicted by the CAMP algorithm to be AMPs (Fig. 2). In order to increase the stringency of the prediction, we further limited these shortlisted AMPs to amphipathic and cationic peptides, since they account for the majority of currently known AMPs^35,36^. 430 peptides from our SCP database satisfied these criteria. We are experimentally validating the antimicrobial properties of these predicted AMPs.

One of these peptides, a 13-mer (IRIILRAQGALKI) derived from vacuolar protein sorting-associated protein 13D-like (Vps13D), demonstrated toxicity against a broad range of Gram-positive and negative bacteria. This peptide was named BING (“Blocker of INter-membrane stress responses of Gram-negative bacteria”) due to its effect on bacterial envelope stress responses (see below). *Vps13D* is a member of a gene family originally identified in yeast and is highly conserved among eukaryotes^37^. While its function is largely unknown^38^, a recent study reported that *Vps13D* could regulate interleukin-6 (IL-6) production in septic shock^39^, which suggests that *Vps13D* may have immunological functions.

### A novel AMP deregulates the components of bacterial envelope stress responses

**Figure 3:**
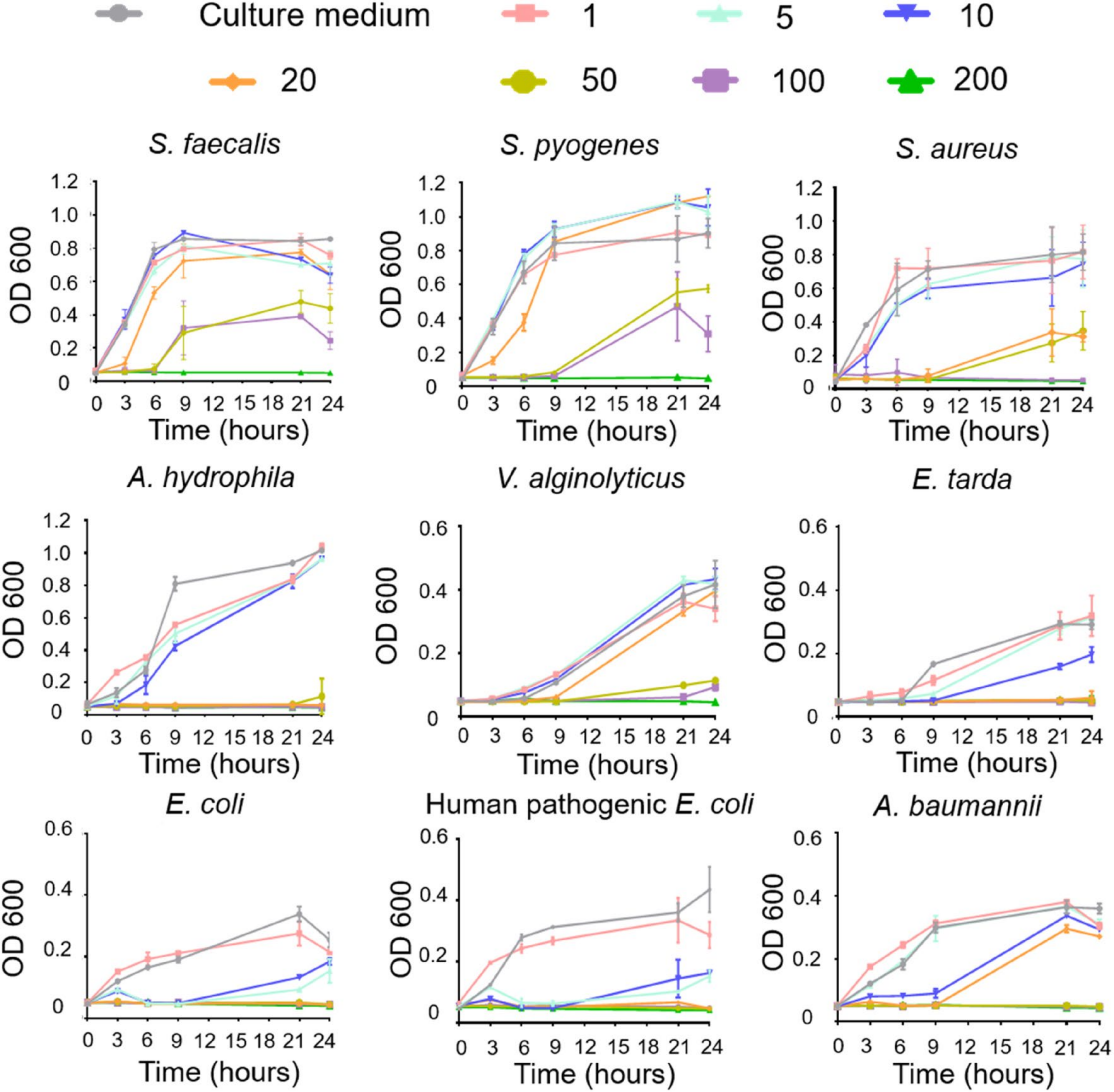
Effect of BING on the proliferation of various bacterial species. Units of peptide concentration: µg/mL. Results are expressed as mean ± SD, n = 3.

**Figure 4:**
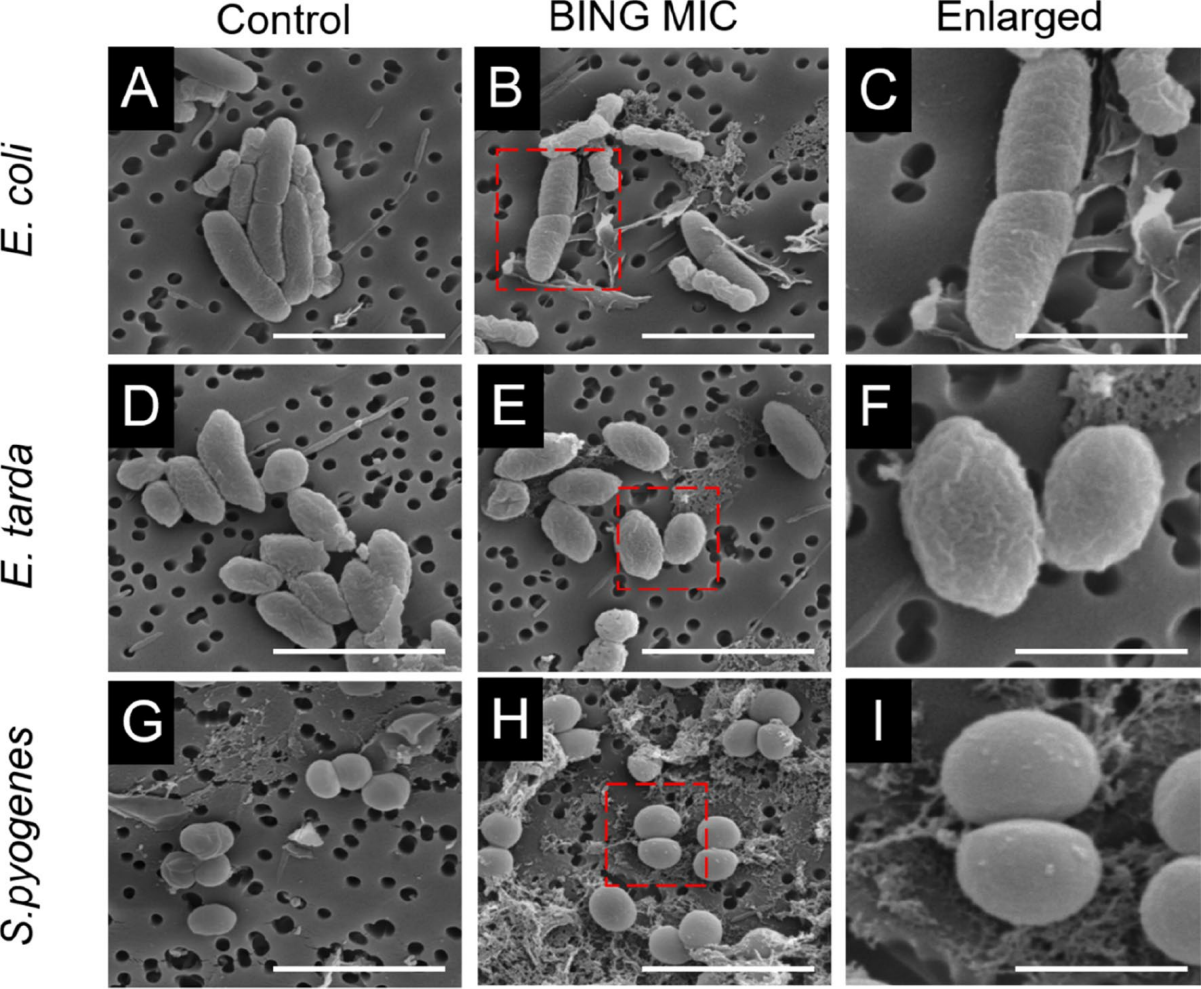
Scanning electron micrographs of *E. coli* (**A**–**C**) *E. tarda* (**D**–**F**) and *S. pyogenes* (**G**–**I**) incubated with BING (7.8 µg/mL for *E. coli*, 10 µg/mL for *E. tarda* and 50 µg/mL for *S. pyogenes*) or culture medium for 1 h. Bars in panels (**A**,**B**,**D**,**E**,**G**,**H**): 3 µm. Bars in (**C**,**F**,**I**): 1 µm. Representative images from three replicates were shown.

**Figure 5:**
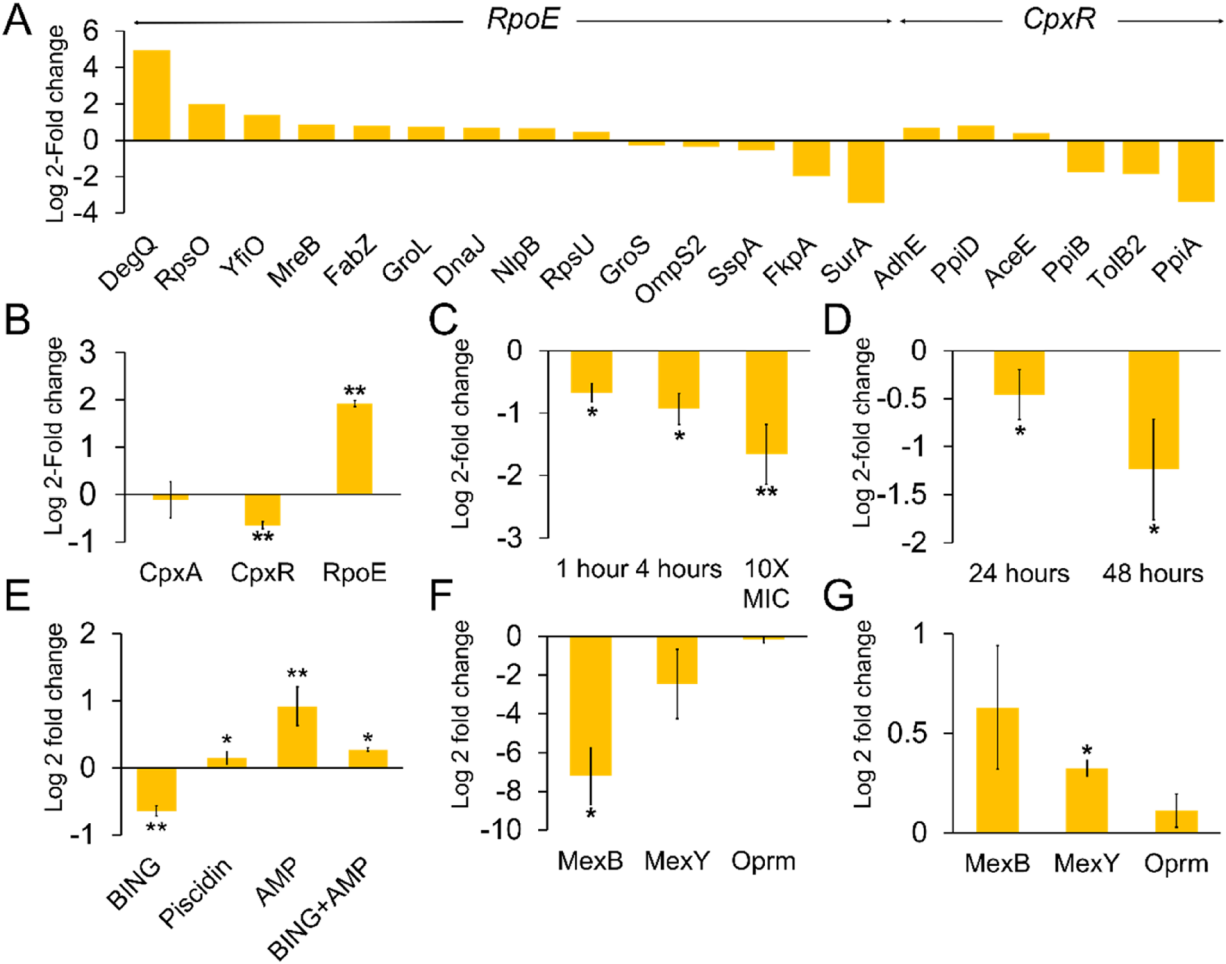
Effect of BING on envelope stress response in Gram-negative bacteria. (**A**) Fold change of selected *E. tarda* proteins after 60 min of BING treatment at 10 µg/mL. (**B**) Relative expression levels of *cpxA*, *cpxR* and *rpoE* in *E. tarda* upon treatment with BING for 60 min at 10 µg/mL. (**C**) *cpxR* gene expression in *E. coli* treated with BING at 10 µg/mL for 1 and 4 h and at 10X MIC (100 µg/mL) for 1 h respectively. (**D**) *cpxR* gene expression in *P. aeruginosa* treated with BING at 25 µg/mL for 24 h and 48 h respectively. (**E**) *cpxR* gene expression in *E. tarda* after the treatment of BING (10 µg/mL), Piscidin (20 µg/mL), Ampicillin (53.1 µg/mL) and BING + Ampicillin (5 µg/mL; 26.5 µg/mL) for 1 h. Expression levels of *mexB, mexY and oprM* in *P. aeruginosa* after the treatment of (**F**) BING (25 µg/mL) and (**G**) Ampicillin (25 µg/mL) for 48 h respectively. All results represented as the mean (n = 3), with error bars indicating SD, of log 2 relative fold change (treatment/control). Statistically significant difference as compared to culture medium controls (*p < 0.05; **p < 0.01, n = 3).

BING showed a broad bactericidal effect (Fig. 3 and Table 1), with MIC (for overnight incubation) ranging from 5 to 50 µg/mL, which is in a similar range to other known AMPs, such as human host defence peptide LL-37^40^. Notably, this peptide was effective against a number of antibiotic-resistant strains, including beta-lactam resistant bacteria (Table 1). The secondary structure of BING was investigated by Far-UV Circular Dichroism (CD) spectroscopy. The CD spectrum in water showed a minimum at 198 nm and an inflexion point at 219 nm, suggesting that this peptide exhibited a random coil conformation in the aqueous environment. As the majority of known AMPs interact with bacterial membrane^6–7^, and as BING could cause significant biochemical changes in bacteria at dosage where there was no observable membrane damage (Figs. 4 and 5), we hypothesised that BING exerted its effect through interacting with the membrane or reaching its intracellular targets through penetrating the membrane. We therefore investigated the CD spectroscopy of BING in a hydrophobic environment. Our data showed that, in 50% TFE, BING displayed prominent changes in the mean residue ellipticity (MRE) at 191 and 215 nm, indicating the formation of a stable secondary structure (Figure S1A). Under this condition, deconvolution analysis revealed a significant β-sheet content up to approximately 40% (Figure S1C). Similar phenomena were also observed in a membrane-mimetic condition containing SDS above the critical micelle concentration (16 mM) (Figure S1B). These results show that this peptide can adopt β-sheet rich conformation depending on the environment, commonly observed in other AMPs^41^. The conformational change associated with peptide-micelle interactions suggests that the bacterial cell membrane may play a role in the folding of this peptide, which might be important for its antibacterial function. A prediction of the 3D structure of this peptide by PEP-FOLD^42^ shows a positively charged hydrophilic patch formed by the clustering of Arg-2, Arg-6 and Lys-12, and a hydrophobic patch composed of Ile-3, Ala-9, Leu-10 and Ile-13 on the opposite surface (Figure S2). It is possible that electrostatic interactions between the positively charged residues and the negatively charged phospholipid head groups of the membrane are involved in the first step of interaction between the peptide and the membrane^43^.

**Table 1:** Antibacterial activity of newly predicted peptide on normal (upper) or drug resistant bacteria (lower).

Bacteria	Gram (+/−)	MIC* (µg/mL)
*Streptococcus Faecalis*	+	50
*Streptococcus pyogenes* ATCC 14289	+	50
*Staphylococcus aureus ATCC 6538*	+	20
*Bacillus subtilis 168*	+	16
*Staphylococcus aureus* ATCC 29213	+	64
*Aeromonas hydrophila ATCC 49140*	−	20
*Vibrio alginolyticus* ATCC 33840	−	50
*Edwardsiella tarda* PE210	−	10
*E. cloacae BAA-1143*	−	32
*Acinetobacter baumannii* ATCC 19606	−	10
*Escherichia coli* ATCC 10536	−	5
*Escherichia coli* (pathogenic)	−	5
*Escherichia coli* BL21 (DE3)	−	8
*Klebsiella pneumoniae* (NDM-1) ATCC BAA-2470	−	32
*Escherichia coli* (NDM-1) ATCC BAA-2469	−	16
NDM-1/BL21 (DE3)	−	4
SHV-1/BL21 (DE3)	−	4
TEM-1/BL21 (DE3)	−	8
MCR-1/BL21 (DE3)	−	16
Methicillin-resistant *S. aureus* ATCC BAA-41	+	32
Multidrug-resistant *S. aureus* ATCC BAA-44	+	32
*Staphylococcus epidermidis ATCC 12228*	+	4
*Pseudomonas aeruginosa* A	−	50

*MIC: the lowest concentration of an antimicrobial agent that can inhibit the growth of bacteria after overnight incubation.

To investigate the bactericidal mechanism of BING, we conducted a quantitative proteomic analysis on *E. tarda* treated with the peptide at 10 µg/mL for 1 h. At this early time point, no observable morphological changes in bacteria were detected (Fig. 4), thus ensuring that any detected proteome change reflected the initial bactericidal action of this AMP and not secondary biochemical changes associated with cell death. We chose *E. tarda*^44^ for this experiment because this marine pathogen^45^ is likely a natural target of fish innate immunity. A total of 770 proteins were identified among three replicates (Supplementary dataset S1), in which 251 proteins were of high confidence (identification in two out of three repeats and supported by more than one peptide). 74 of these proteins were found differentially expressed (at least one-fold difference) in response to the AMP (Supplementary dataset S2). The proteins involved in outer membrane assembly, peptidoglycan synthesis, and metal ion binding were induced, possibly representing cellular defences against BING toxicity. Interestingly, many members of the Peptidyl-prolyl isomerase (PPIases) family were highly suppressed, e.g., peptidyl-prolyl *cis*–*trans* isomerase (*surA*)^46,47^, peptidyl-prolyl *cis*–*trans* isomerase A (*ppiA*) and peptidyl-prolyl *cis*–*trans* isomerase B (*ppiB*), and FKBP-type peptidyl-prolyl *cis*–*trans* isomerase (*fkpA*)^48^ (Fig. 5A). These proteins are all involved in the homeostasis of periplasmic proteins in Gram-negative bacteria^49^. Periplasmic proteins are highly sensitive to proteolysis and aggregation and therefore often require chaperone proteins to prevent proteolysis and aggregation^50^. The suppression of *surA* and other PPIases by this AMP possibly leads to the accumulation of misfolded proteins in the periplasmic compartment. Interestingly, trypsin-like serine protease (*degQ*), a protein responsible for degrading misfolded proteins^51^, was extensively increased in the treated cells, suggesting the need to remove these misfolded periplasmic proteins. It will be interesting to investigate whether knocking down *degQ* expression could sensitise bacteria to BING. Proteome analyses on the effect of other cationic AMPs on Gram-negative bacteria have been conducted^3,52^, but deregulation of proteins involved in envelope stress responses have never been reported, suggesting that this AMP exhibits a unique antibacterial mechanism. We thereby named this AMP “Blocker of INter-membrane stress responses of Gram-negative bacteria” (BING).

### BING downregulates the expression of *CpxR* in Gram-negative bacteria

Periplasmic stress responses in bacteria are mainly regulated by *rpoE* signalling and the CPX two-component pathway^49,53^. We characterized the mRNA levels of *cpxR* and *rpoE* in *E. tarda* treated with BING at 10 µg/mL for 1 h. As shown in Fig. 5B, the expression of *cpxR*, one of the components in the CPX two-component system, is moderately but significantly reduced by BING treatment. *cpxA*, the other component of this system, remains unaffected. *cpxR* and *cpxA* are adjacent genes encoded by the same *cpxRA* operon^54^, suggesting that BING effect on *cpxR* may be post-transcriptional. The apparent differential regulation of the two components of the *cpxRA* system by BING is an interesting observation that warrants future investigation. By contrast, the expression of *rpoE* was significantly induced by BING. This is consistent with the upregulation of *degQ*, *rpsO* and *yfiO*, which are the downstream proteins of the *rpoE* pathway, as shown in our proteomic data (Fig. 5A). The downregulation of *cpxR* expression was also observed in *E. coli* and *P. aeruginosa,* in dose-dependent manner (Fig. 5C,D). As *cpxR* is not an essential gene^55^ (Ref.^54^ and Supplementary figure S4), the effect of BING on the expression of *cpxR* is unlikely the direct cause of BING-mediated cell death. It is possible that a prolonged treatment of BING may affect additional pathways, which ultimately led to the destruction of cell walls (Supplementary figure S3). However, as *cpxR* is pivotal to many aspects of bacterial stress responses, including AMR, we focused our research on the functional consequences of *cpxR* suppression during the initial phase of BING treatment.

As far as we know, BING represents the first AMP that suppresses the expression of *cpxR,* and possibly modulates the periplasmic stress response in Gram-negative bacteria. Piscidin, a known fish AMP, has no significant effect on the level of expression of *cpxR* (Fig. 5C). Another AMP, ApoEdpL-W, has been reported to trigger the *cpxAR* stress pathway^56^. Ampicillin significantly induced the expression level of *cpxR* (Fig. 5E), consistent with the reported effect of antibiotics on *cpxR*^57^ and on envelope stress response^58^. Notably, BING could revert the induction of *cpxR* expression by ampicillin (Fig. 5E). As we aimed to explore the potential applications of BING against human diseases, *E. coli* and *P. aeruginosa* were used as model organisms in our subsequent studies.

### BING downregulates efflux pump components and synergises the effect of antibiotics

**Figure 6:**
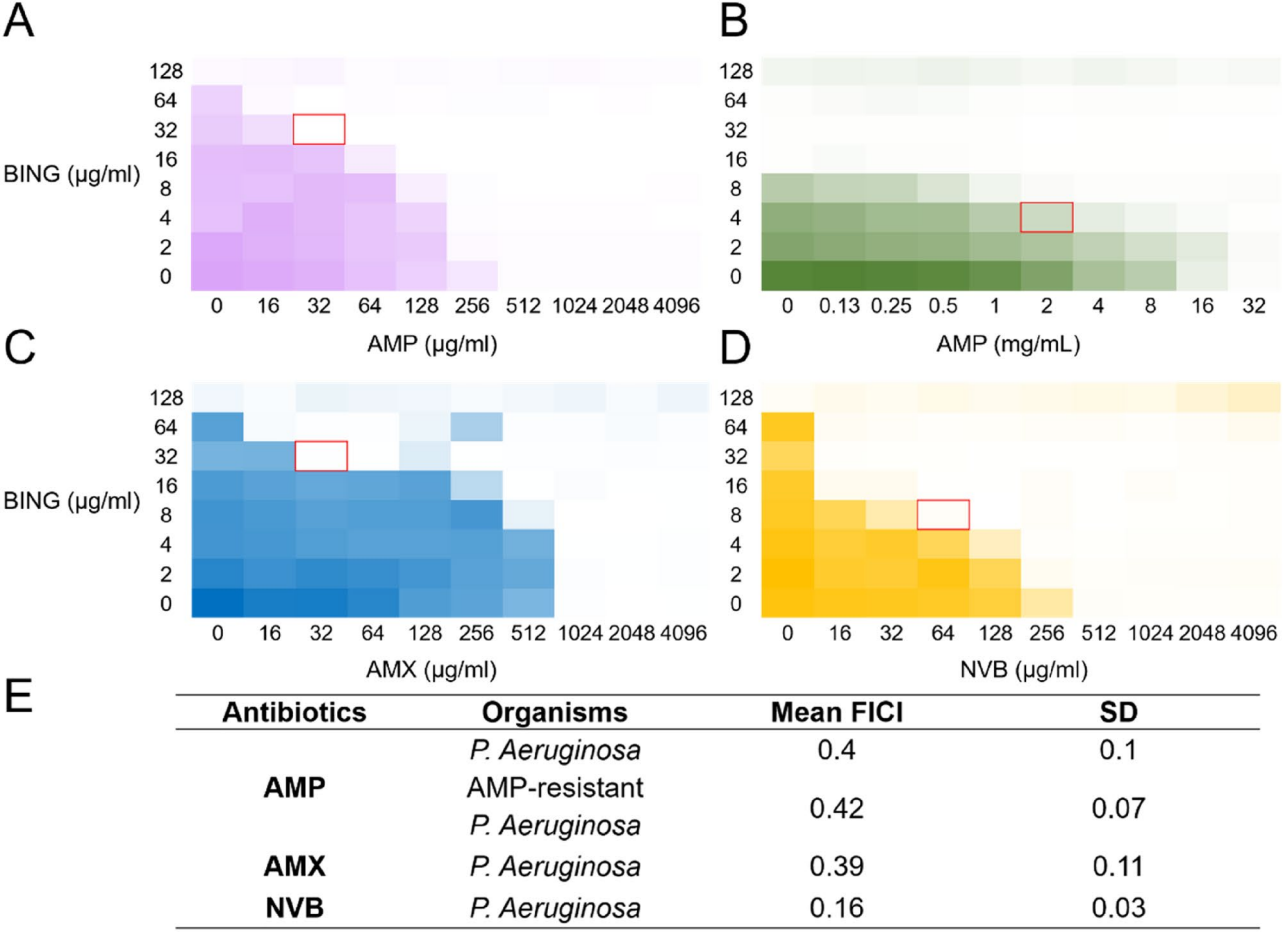
Combinational effect of BING and antibiotics. Checkerboard assay on the growth of (**A**) *P. aeruginosa* and (**B**) ampicillin-resistant *P. aeruginosa* treated with BING and Ampicillin. (**C**,**D**) Checkerboard assay on the growth of *P. aeruginosa* with BING and Amoxicillin or Novobiocin, respectively. Representative data from three replicates. (**E**) Summary of FICIs of all the checkerboards. *AMP* ampicillin, *AMX* amoxicillin, *NVB* novobiocin.

*cpxR* is involved in many aspects of AMR development^59,60^. In *K. pneumoniae*^61,62^ and *V. cholerae*^63^, the activation of drug efflux pump has been shown to be *cpxR*-dependent. In particular, the over-expression of *cpxR* is associated with the activation of the *mexA* promotor, hence upregulating the *MexAB-OprM* efflux pump in *P. aeruginosa*^64^. We tested if the downregulation of *cpxR* caused by BING could lead to a change in the expression of efflux pump components. As shown in Fig. 5F, the expression of transporter genes *mexB*, *mexY,* and, to a lesser extent, *oprM* in *P. aeruginosa* was significantly reduced after the exposure to BING. By contrast, ampicillin, which upregulates *cpxR* (Fig. 5E), induced the expression of these genes (Fig. 5G). Next, we asked whether the suppression of efflux pump expression in *P. aeruginosa* by BING could sensitise these bacteria towards antibiotic toxicity. Two-dimensional checkerboard experiments were conducted to test the combinational effects of BING and ampicillin (Fig. 6A). Our data show that the presence of BING could significantly enhance the effect of ampicillin (Fractional Inhibitory Concentration Index (FICI) of 0.4). E.g., the MIC of ampicillin was decreased by eightfold in the presence of 32 µg/mL of BING. Similar synergistic effects were also observed with amoxicillin (FICI 0.39), another beta-lactam, and with novobiocin (FICI 0.16), an aminocoumarin (Fig. 6C–E). The synergism between BING and ampicillin was also observed in *P. aeruginosa* previously selected for ampicillin-resistance (FICI 0.42, Fig. 6B). Notably, in the presence of 8 µg/mL BING, the MIC towards ampicillin in the ampicillin-resistant bacteria was reverted to the wildtype level, suggesting that BING may potentially restore the antibiotic sensitivity of drug resistant bacteria.

### BING suppresses the development of antibiotic resistance

**Figure 7:**
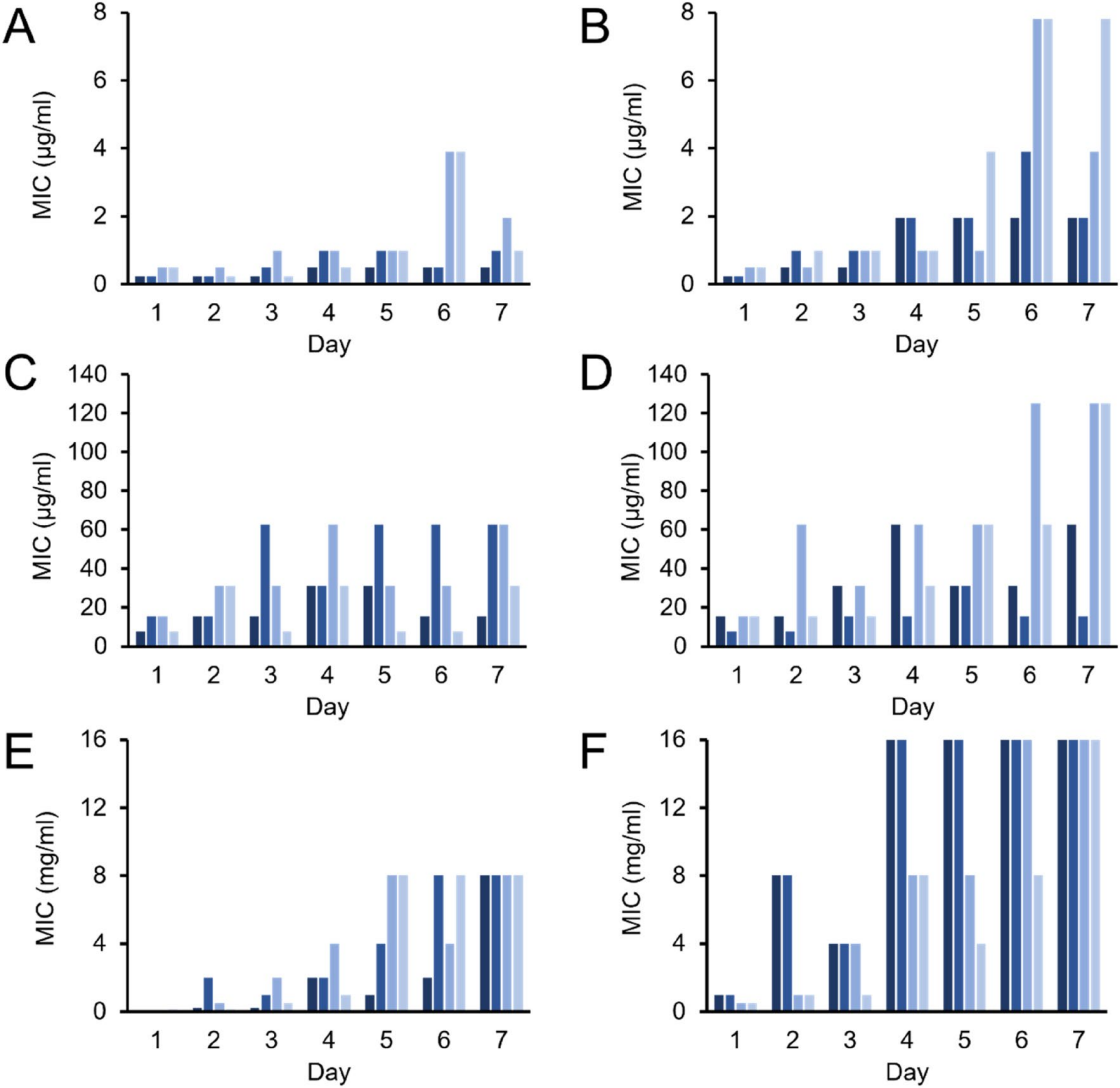
Effect of BING on the development of antibiotic resistance. Each bar represents one of the four replicates in each experiment. Bacteria (panels **A**–**D**: *E. coli*, panels **E**,**F**: *P. aeruginosa*) were cultured in an increasing concentrations of antibiotics and cells survived at the highest antibiotic concentration were passaged daily for 7 days. Data here show the MIC of the selected cells on each day. (**A**) *E. coli* treated with kanamycin in the presence of BING (3.9 µg/mL). (**B**) *E. coli* treated with kanamycin alone. (**C**) *E. coli* treated with ampicillin in the presence of BING (3.9 µg/mL). (**D**) *E. coli* treated with ampicillin alone. (**E**) *P. aeruginosa* treated with ampicillin in the presence of BING (35 µg/mL). (**F**) *P. aeruginosa* treated with ampicillin alone.

The deletion of *cpxR* in *E. coli*^65^, *S. typhimurium*^66^ and *E. amylovora*^67^ has been shown to increase sensitivity towards beta-lactams. Mutations of *surA*, one of the PPIases that was also suppressed by BING, can increase the bacterial sensitivity to antibiotics due to defects in the membrane integrity^47^. As BING reduced the expression and the ampicillin-induced upregulation of *cpxR* (Fig. 5E,G), we asked if this AMP could attenuate the development of AMR. To develop AMR, *E. coli* were passaged daily in increasing titres of kanamycin or ampicillin for 7 days, resulting in an increase of MIC of kanamycin by at least 8 folds and ampicillin by at least 4-folds (Fig. 7B,D). In the presence of a sublethal dose of BING (3.9 µg/mL) during these 7 days, the increase of MIC of both antibiotics were significantly decreased (Fig. 7A,C), suggesting that the exposure of BING could reduce the development of AMR. Similar results were observed in the development of ampicillin resistance in *P. aeruginosa* (Fig. 7E,F). Accordingly, the deletion of *cpxR* also led to a similar retardation of ampicillin resistance in *E. coli* (Supplementary figure S4). These data show that BING not only synergises with antibiotics but, at sublethal concentrations, it reduces the rate of resistance development.

### Low toxicity of BING towards mammalian cells and fish

**Figure 8:**
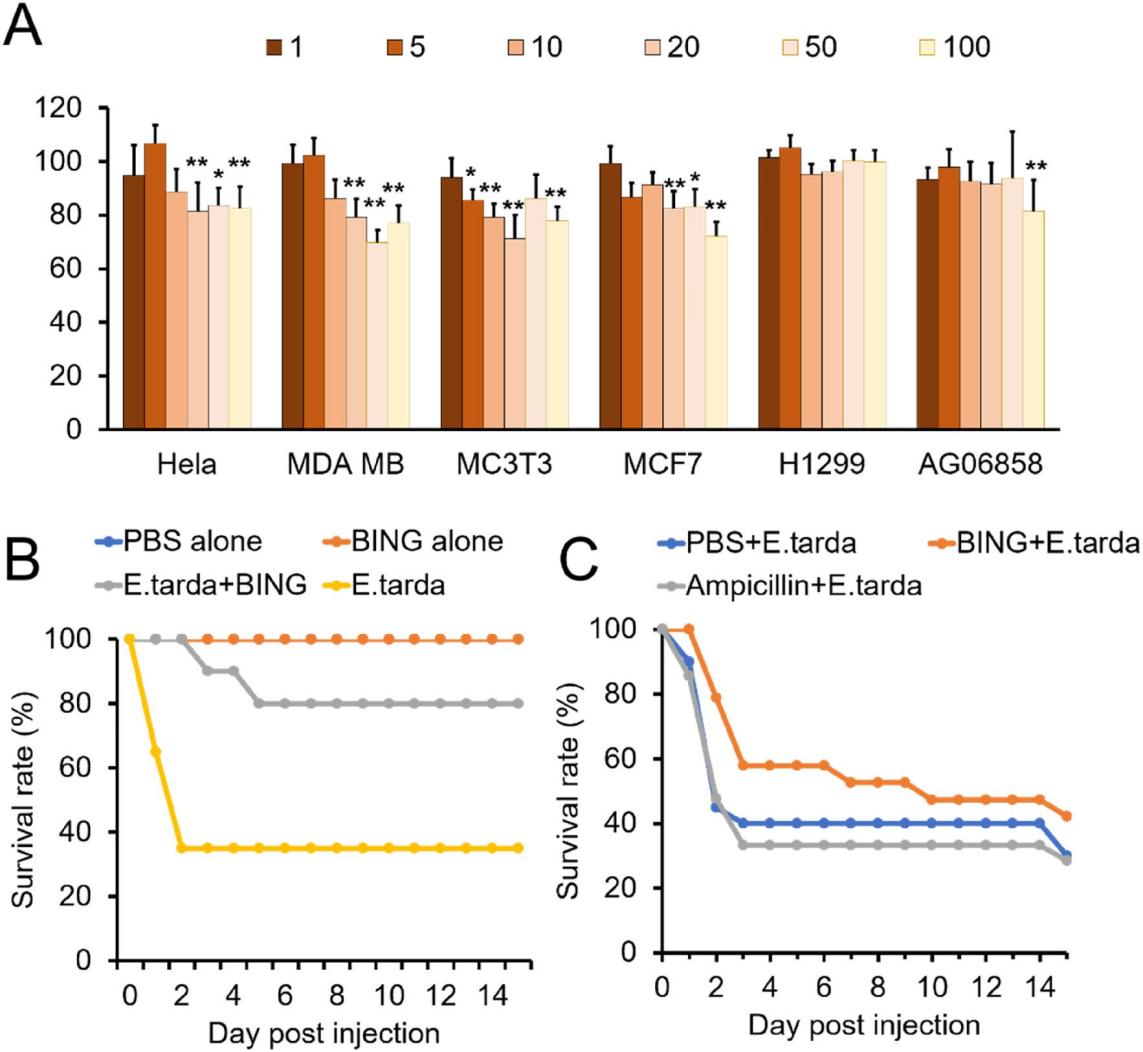
Effect of BING on mammalian cells and medaka fish. (**A**) Effect of BING on the viability of cultured mammalian cells, incubated for 48 h in the presence of BING at different concentrations (µg/mL), as determined by MTT assay. Cell viabilities are represented as peptide treated cell numbers over medium-only control. Statistically significant difference as compared to controls (*p < 0.05, **p < 0.01, n = 4). (**B**,**C**) Survival of medaka fish injected with a lethal dose of *E. tarda* (10^4^ CFU/fish) with or without AMP and antibiotic. (**B**) Co-injection experiment: BING only, injected with 1 µL of BING (1 mg/mL); *E. tarda* + BING: injected with 1 µL of *E. tarda* (10^7^ CFU/mL) and BING (1 mg/mL) mixture; *E. tarda* alone (1 µL of *E. tarda*: 10^7^ CFU/mL). (**C**) Sequential injection experiment: 1 µL of BING (2 mg/mL), Ampicillin (2.1 mg/mL) or PBS injection followed by *E. tarda* injection after two hours (1 µL of *E. tarda*: 10^7^ CFU/mL). 20 fish per group. Log-rank (Mantel-Cox) test was applied for the comparison of survival curves (*p < 0.05; **p < 0.01).

The cytotoxic effect of BING was tested against a panel of human cancer and primary cell lines (Fig. 8A). Relative low toxicity was detected after 48 h of treatment (at least 32 h longer than the duration used for MIC measurements in bacteria), at concentrations comparable to the MIC for gram-negative bacteria. In addition, no adverse effect was observed in adult Medaka fish intraperitoneally injected with 1 µg of the peptide for up to 14 days (Fig. 8B).

Next, we asked whether BING could protect Medaka from bacterial infection. We injected *E. tarda* bacteria into medaka with or without BING. As shown in Fig. 8B, medaka survival was significantly higher (80%) in fish co-injected with BING and bacteria, compared with the ones without BING treatment (35%). We then tested whether medaka could be protected from bacteria-induced lethality by BING. Fish that had been injected with BING were subsequently challenged by *E. tarda*. Control groups were either pre-injected with PBS or with Ampicillin. Figure 8C shows that the initial survival rate (1 day post injection) in BING group (85%) was higher than the other two groups (about 45%). Interestingly, ampicillin appeared ineffective in treating *E. tarda* infection in medaka. Taken together, the treatment of exogenous BING appears to alleviate the effect of *E. tarda* infection on medaka.

### C-terminus amidation and d-amino acid substitution increased the stability of BING without compromising its antibacterial activity

**Figure 9:**
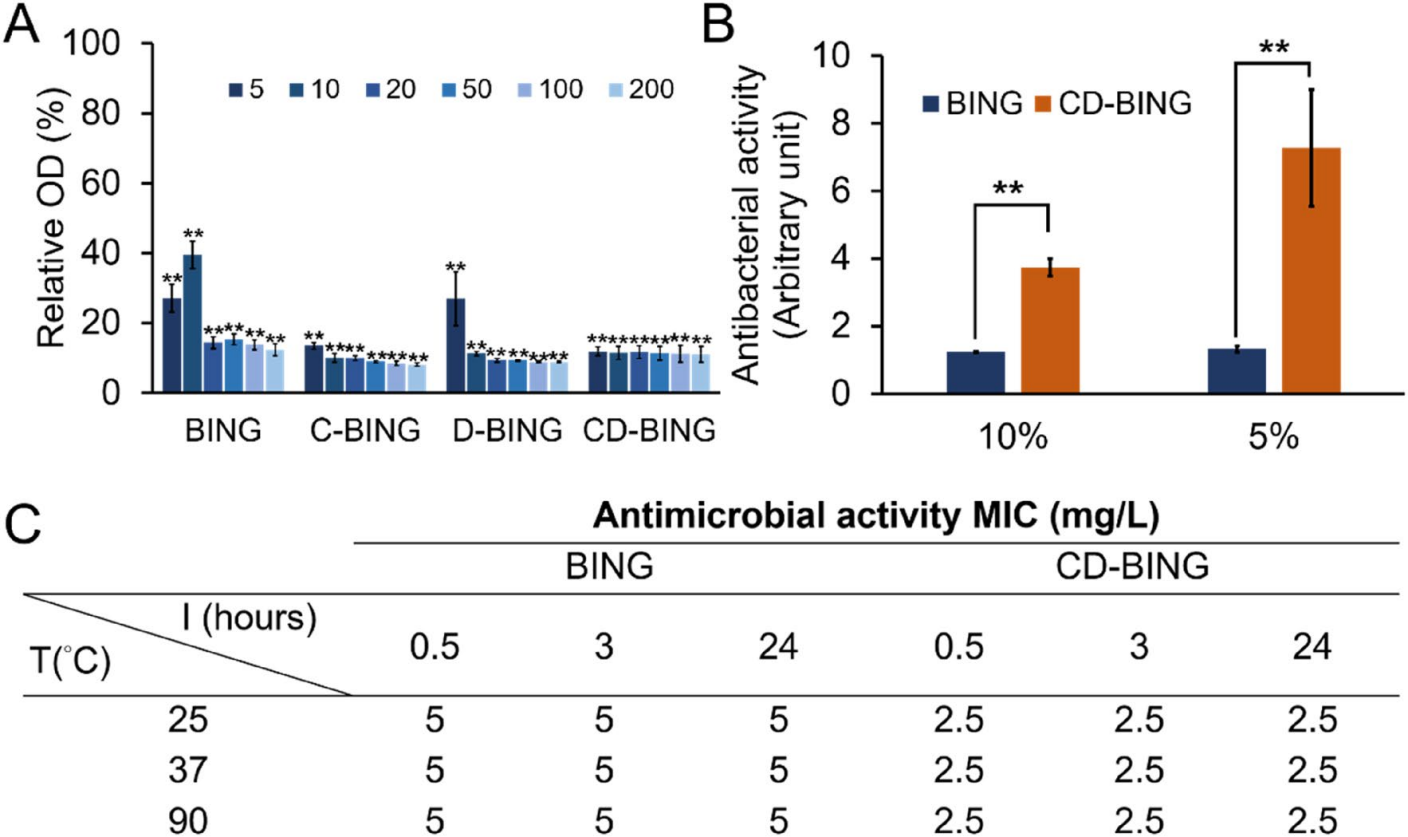
Antibacterial activities of modified BING. (**A**) Relative viability of *E. coli* treated with BING that is unmodified (BING), C-amidated (C-BING), d-isomer substituted (D-BING) and both C-amidated and d-isomer substituted (CD-BING) for 16 h. Data are expressed as relative cell density compared to the untreated group. Unit of peptide concentration: µg/mL. (**B**) MIC of BING and CD-BING against of *E. coli* in the presence of 5% and 10% of fetal bovine serum. Data are expressed as the MIC relative to those of BING and CD-BING in the absence of serum. Bars represent means (n = 3) with error bars indicating SD. The statistically significant difference as compared to culture medium controls (*p < 0.05; **p < 0.01). (**C**) MIC of BING and CD-BING pre-incubated at different temperatures for 0.5, 3 and 24 h (n = 3).

The interesting antibacterial properties of BING make it a promising lead compound for clinical translation. However, linear AMPs are often undesirable as drug candidates due to their relative instability. Chemical modifications of peptides, such as C-terminus amidation and d-amino acid substitution, are known to increase peptide stability in vivo^68,69^. We tested if these modifications could affect the antibacterial effect of BING. As shown in Fig. 9A, C-terminus amidation (C-BING), complete d-amino acid substitution (D-BING) and the double modification (CD-BING) of BING did not affect its antibacterial activity (Fig. 9A). In fact, these modifications led to a slight increase in BING toxicity, possibly as a result of the enhanced stability. In addition, CD-BING was more resistant than unmodified BING to the action of fetal bovine serum (Fig. 9B). Finally, we tested the thermostability of BING and CD-BING (Fig. 9C). Our data show that the activity of both peptides against *E. coli* remained unaffected after a pre-exposure at 25 °C, 37 °C and 90 °C for up to 24 h. This level of thermostability exceeds that of other AMPs in similar studies^70,71^.

In conclusion, our proteomic characterisation of Medaka plasma peptides have led to the discovery of BING, a novel AMP that downregulates the expression of *cpxR*, which plays a central role in the survival mechanism of bacteria exposed to stress. As far as we know, BING is the first molecule reported to suppress the expression of *cpxR* and is therefore an attractive lead compound as a *cpxR* inhibitor. As BING and its derivative CD-BING are thermostable and showed relatively low toxicity to fish and mammalian cells at its bactericidal concentrations, this AMP is a promising candidate for antimicrobial intervention or even as food preservative^72^. Future investigations will be focused on the molecular mechanism that underlies BING’s effect on *cpxR* synthesis and on in vivo studies on the safety and antibacterial actions in mammals. The other AMP candidates identified by this study will be a rich resource for the discovery of novel antibacterial compounds.

## Materials and methods

### Cells and animals

The bacterial strains used in this study are listed in Supplementary table S1.

HeLa, MCF7, MDA-MB, H1299 and MC3T3 E1 cells were purchased from American type culture collection (ATCC^®^ CCL-2™, ATCC^®^ HTB-22™, ATCC^®^ CRM-HTB-26™, ATCC^®^ CRL-5803™, ATCC^®^ CRL-2593™). AG06858 was obtained from Coriell Institute (Coriell cell collection). The details of mammalian cell lines used in this study are listed in Supplementary table S2. All of the mammalian cells were cultured in low glucose Dulbecco’s modified eagle medium (DMEM) (Invitrogen) supplemented with 10% (v/v) fetal bovine serum (Gibco), 1% (v/v) Antibiotic–Antimycotic (Invitrogen) and 1% (v/v) Glutamax (Invitrogen) in a humidified incubator supplied with 5% CO_2_. The medium was changed every 3 days and the cells were sub-cultured when they reached 80% confluence. Japanese medaka (*Oryzias latipes*) were maintained at the City University of Hong Kong. The water temperature was maintained at 26 ± 0.5 °C and the light: dark cycle was adjusted to 14 h:10 h. Water was replaced every 2 days with dechlorinated tap water. Fish were fed twice daily with lake food and once with *Artemia nauplii*. This study was carried out in compliance with the ARRIVE guidelines for the involvement of animals. All animals were treated according to the guidelines released by the European Union EU Directive 2010/63/EU for animal experiments. The experiment has been reviewed and approved by the City University of Hong Kong Research Committee for its compliance with the Animals (Control of Experiments) Ordinance, Cap. 340, of the Hong Kong Special Administrative Region.

### Collection of peptides from *O. latipes* plasma

Plasma was collected from 32 healthy and bacterially infected medaka fish of both sexes and different ages (see Fig. 1 for details). Bacterial infection with *E. tarda* was performed as previously described^73^. Plasma from healthy and 3 day post infected fish was collected with syringes that contained a mixture of heparin (500 i.u/mL) and protease inhibitors (ROCHE complete protease Inhibitor Cocktail tablets) in phosphate buffered saline (PBS)^74^ after anesthetization of the fish with 0.04 mg/mL of ethyl-3-aminobenzoate methanesulfonate salt (MS-222; Sigma-Aldrich E10521, St. Louis, MO). To obtain small peptides, plasma were diluted in 25 mM ammonium bicarbonate (ABC) and 20% (v/v) acetonitrile (ACN) with 1:5 ratio to dissociate protein complexes in the plasma^33,75^. After that, an Ultrafilter was used to fractionate peptides under 3 kDa in accordance with the manufacturer’s instructions (Amicon Pro Purification System with 3 kDa Amicon Ultra-0.5 Device, ACS500302, Merck Millipore Ltd.). Filtrates were collected and concentrated by freeze-drying. The concentrated peptides were dissolved in 2% ACN with 0.1% TFA and then separated through high-performance liquid chromatography (HPLC; Dionex) on a C18 reverse phase column (inner diameter 75 μm, 5 μm of Acclaim PepMap100 medium; Dionex) over an 180 min gradient (mobile phase A: 0.1% fluoroacetic acid (FA) in 2% acetonitrile (ACN) in MilliQ water, mobile phase B: 0.1% FA in 98% ACN) and analysed by a mass spectrometry MS/MS system (µTOFQII; Bruker Daltonics), as shown in Fig. 1. The resulting peak lists were generated by using Data Analysis, Version 4.0 (Bruker Daltonics). The MS data were searched against the NCBI database for *Oryzias latipes* (1411 release, 24,495 sequences searched), by using the MASCOT search engine (Matrix Science 2.3.02). The fixed modification was set as carbamidomethyl (C) and variable modification used was oxidation (M). No enzyme specificity was set and peptide charges of + 2 and + 3 were selected. Only ions with Mascot scores higher than 57 were taken into consideration. The identified fish circulating peptides were compared against the 2767 experimentally validated AMPs curated in CAMP_R3_ by using BLASTP. A peptide was defined as highly homologous to a known AMP if the E-value of the BLASTP search was lower than 0.05, or if the amino acid sequences of the two peptides were at least 80% identical over 75% of the length of at least one of the sequences.

### AMP prediction

The identified fish circulating peptides were blasted against around 2700 experimentally validated AMPs obtaining from CAMP_R3_ [(http://www.camp.bicnirrh.res.in/) by using BlastP provided by NCBI. The results either with identities higher than 80% (with coverage higher than 75%] or *E* values lower 0.05 are defined as highly homologous with known AMPs. The remaining fish peptides were interrogated against the CAMP_R3_ database^34^ where each peptide was scored for likelihood of predicted antimicrobial property. Three algorithms provided by CAMP_R3,_ including Support Vector Machine (SVM), Random Forest (RF) and Discriminate Analysis (DA), were applied with probability values. At least one of the probability values higher than 0.5 were regarded as novel AMPs. Length, charge and hydrophobic residues of the peptides were calculated by using the APD2 database properties calculator^5^. Molecular weights and isoelectric points were calculated using ENDMEMO (http://ww.endmemo.com/bio/proie.php) and their hydrophobicity (GRAVY) was calculated using GRAVY (http://www.gravy-calculator.de/).

### Peptide synthesis

The peptides were synthesized through a conventional Fmoc-based solid-phase peptide synthesis in accordance with previously established protocols^76^. In brief, the peptides were synthesized on Rink amide resin. Fmoc-deprotection was carried out with 20% piperidine in dimethylformamide (DMF) while amino acid building blocks were coupled to the resin by using HOBT/HBTU/DIEA. C-terminal amidated peptide was synthesized as amide and capped in the amino terminal with acetic anhydride^77^. The peptides were then cleaved with a mixture of TFA/TIPS/H_2_O/EDT and characterized through MALDI–TOF–MS (Bruker Daltonik GmbH, Bremen, Germany) as previously described^78^. The peptides were purified by using a Shimadzu (Shimadzu Corporation, Japan) liquid chromatograph, equipped with a vacuum degasser, a binary pump, and a diode array detector (DAD) system, powered by Empower3 software. A reverse-phase Phenomenex Luna 5 μm C18 (2) 100 Å 50 × 3.0 mm column was used for separation at a column temperature of 30 °C. The samples were separated by using a gradient of the mobile phase which contained Solvents A (acetonitrile) and B (0.1% trifluoroacetic acid (TFA) in water) with the following elution programme: 0–3 min, 5–10% A; 3–15 min, 10–75% A; 15–18 min, 75–95% A; and 18–20 min, 95–5% A. The detection wavelengths were set at 215 nm and 254 nm. The flow rate was set at 5.0 mL/min and the injection volume was 500 µL. The purified peptides (1.0 mg/mL) were dissolved in water or acetonitrile, and characterized by MALDI–TOF–MS (Bruker Daltonik GmbH, Bremen, Germany)^78^.

### Circular dichroism spectroscopy

Far-UV CD spectra were collected at room temperature using a Jasco J-810 spectropolarimeter (Jasco, Tokyo, Japan). BING peptide was dissolved in DI water to make a stock solution (1.8 mM). CD samples were prepared by diluting the peptide stock into trifluoroethanol (TFE)-H2O mixtures and Sodium dodecyl sulfate (SDS)-H2O mixtures with increasing ratio of TFE from 0 to 50% and SDS from 0 to 16 mM, respectively. The spectra were recorded with a scanning speed of 100 nm/min at wavelength from 190 to 280 nm. For all experiments, the reference spectrum of the respective solvent was monitored to serve as the background and the data were then subtracted from the corresponding CD spectrum. All spectra were averaged over five scans and converted to mean residue ellipticity [θ]. The secondary structure of all were estimated by using BESTSEL (http://bestsel.elte.hu/)^79^. Three-dimensional (3D) structure of the peptide was created using PEP-FOLD tool and visualized by the PyMol programme (DeLano Scientific). The electrostatic surface charge distribution was calculated using the ABPS plugin in PyMOL.

### Antimicrobial assay

Bacteria were grown in their respective media (Supplementary table S1) overnight at 37 °C or room temperature with constant shaking and then diluted to 10^6^ CFU/mL in their respective medium. Bacteria and peptide were incubated (50 µL: 50 µL) to make the final peptide concentrations (1, 5, 10, 20, 50, 100, 200 µg/mL) in a 96-microtitre plate and incubated for 24 h with constant shaking. The control wells had the same number of bacteria without the peptide. Absorbance at 600 nm was detected every 3 h up to 24 h to obtain the growth curves. The minimal inhibition concentration (MIC) was defined as the lowest concentration of an antimicrobial agent that could inhibit the growth of bacteria after overnight incubation^80^.

### Scanning electron microscopy

*E. tarda, E. coli* and *S. pyogenes* cells (10^6^ CFU/mL) were incubated with 6.8 and 50 µg/mL of BING or culture medium for 30 min and then collected on a polycarbonate filter (pore size of 0.2 µm). The cells were fixed in 10% (v/v) glutaraldehyde diluted in PBS (pH 7.2) for 24 h at 4 °C. The samples were viewed with a Philips XL30 ESEM FEG environmental scanning electron microscope (Philips Electronics, Netherlands, Europe) operated at 10 kV.

### Proteomic characterisation of BING-treated bacteria

*E. tarda* treated with BING (10 µg/mL for 1 h) and the untreated control were separately washed and lysed in 8 M of urea for 2 h. The lysate was then reduced in 10 mM of dithiothreitol (DTT) in 50 mM of ammonium bicarbonate (30 min at room temperature) and alkylated in 50 mM of iodoacetic acid (IAA) in 50 mM of ammonium bio- carbonate (20 min at room temperature). The samples were diluted 8 times with ammonium bicarbonate (50 mM, pH 8.0) so that the final concentration of urea was 1 M, followed by 2 μg of trypsin (Roche, Mannheim, Germany) for digestion. The mixtures were then incubated overnight at 37 °C and dried in a vacuum centrifuge (Eppendorf Concentrator plus). The peptides were dissolved in 20 μl of 0.1% trifluoroacetic acid (TFA), followed by ZipTip (Millipore, Billerica, MA, USA) purification that was carried out in accordance with the manufacturer’s instructions.

Mass spectrometry (MS) data were collected on an Orbitrap Fusion mass spectrometer coupled online with a nanoUPLC EASY-LC-1000 liquid chromatography system (ThermoFisher Scientific™, San Jose, CA). The peptides were eluted by using a microcapillary column with 50 cm × 75 µm ID, PepMap C18, 2 µm particles (ThermoFisher Scientific™) with 2 to 80% acetonitrile in 0.1% formic acid at a flow rate of ~ 350 nL/min for 120 min of gradient. They were ionized through electrospray ionization (positive mode 2.1 kV), and survey scans of peptide precursors from 350 to 1550 m/z were performed at a resolution of 120 K at 200 m/z. Tandem MS was performed by isolation at 1.2 Th with the quadrupole component, fragmentation with normalized collision energy of 35%, and rapid scan MS analysis in the ion trap. The MS2 ion count target was set to 10E4 and the maximum injection time was 250 ms. Only precursors with a charge state of 2–7 were fragmented for the MS2 analysis. The dynamic exclusion duration was set to 60 s with a 10 ppm of low and high mass tolerance around the selected precursor and its isotopes. The instrument was run in data dependent mode by using Orbitrap and an ion trap with − 3 s cycles.

The raw data were analysed by using Proteome Discoverer (Version 1.4.0.288. Thermo Fisher Scientific). The MS/MS spectra were searched with the Mascot search engine against the NCBI bacteria database 2014 (with 29,574 sequence entries). The peptides were generated from a Lys-C and tryptic digestion and the precursor mass tolerance was set as 25 ppm, and 0.6 Da for fragment mass tolerances. The oxidation of methionine and deamidation of N, Q were set as the dynamic modification, and the carbamidomethylation of cysteine-C as the static modification. The peptide spectral matches (PSMs) were validated by using a percolator based on q-values at a 1% false discovery rate (FDR). For protein identification, a high-confidence database search with a peptide target FDR (strict) of 0.1%, and target FDR (relaxed) 0.5% and ranked one peptide was used for peptide filtering. Identified peptides were grouped into individual protein clusters by scaffolding.

Peptide quantitation was carried out by using Progenesis LC–MS (version 4.1, Nonlinear) and as previously described^81^. In brief, the profile data of two sample groups with triplicates for each were converted into MS features. After “Automatic Alignment”, all features with a charge that ranged from + 2 to + 7 were subjected to normalization. Following a statistical analysis, processing was carried out by using transformed “log-like” arcsin (ANOVA) calculations of all the detected features. The total cumulative abundance was calculated by summing the abundance of all peptides that belonged to a respective protein. The proteins were then ranked by *p*-value (one-way ANOVA) based on the sum of the normalized abundance across all runs.

### Adaptive evolution of antibiotic resistant *P. aeruginosa* and *E. coli*

*P. aeruginosa* and *E. coli* were evolved in ampicillin, with or without the sublethal concentration of BING, over a 7-days course by broth dilution method. Six different overnight cultures of bacteria were diluted to 10^6^ CFU/mL individually. 100 µL of diluted bacteria culture was treated with various concentrations of ampicillin ranging from 3.8 to 250 µg/mL to make a final volume of 200 µL in each well of a 96 well-plate. After 18–22 h incubation, OD was determined by the spectrophotometer. The wells with the highest antimicrobial agents concentration which showed the distinct growth (OD_600_ > 0.1) in the 96-well plates were chosen to inoculate a fresh drug gradient. The procedure continued for 7 days and some resistant bacteria were stored in 25% glycerol for further study.

### Checkerboard assays

Two-dimensional checkerboard assays were performed to evaluate the synergistic effect of BING and other antibiotics in wild type and ampicillin-resistant *P. aeruginosa*. Briefly, various concentrations of BING and antibiotics with two-fold serial dilution were added into the 96-well plate vertically and horizontally, respectively. 10^6^ of bacteria were added into each well of 96 well plates containing the combined antimicrobial agents. After 18–22 h incubation for 37 °C at 250 rpm, OD_600_ was determined by the spectrophotometer to indicate the growth of bacteria. Three replicates were performed.

### Quantitative real-time PCR

The total RNA were collected from *E. tarda, P. aeruginosa* or *E. coli* (10^6^ CFU/mL) by using a RNAprep pure cell/bacteria kit (TIANGEN), then reverse-transcribed into cDNA by using the One-Step FastQuant RT reagent kit with gDNase (TIANGEN). 18S ribosomal RNA (18S) was used as a reference gene for qPCR normalization^82^. Each group contained 6 biological replicates. Quantitative polymerase chain reaction (qPCR) assays were performed by using fluorescent dye powder SYBR Green PCR Master Mix and the ABI 7500 System. Gene-specific primers were designed with Primer-BLAST (NCBI). Specific primers were designed based on the sequence of the mRNA by the NCBI blast primer software. The primers used in the detection are listed in Supplementary table S3. The relative expression levels (fold change) of the tested genes were calculated by using the relative expression based on the 2^(−Delta Delta C (T))^ method.

### In vitro cytotoxicity assay

Mammalian cell lines (Hela, MDA MB, MC3T3, MCF7, H1299, and AG06858) were seeded into 96 well-plates 1 day before treatment at a density of 3 × 10^5^ cells/mL for a MTT assay. The peptides were directly dissolved in the medium to make a 200 µg/mL stock. After incubation with different cell lines in the 96-well plates for 48 h, 5 mg/mL MTT (3-(4,5-Dimethylthiazol-2-yl)-2,5-Diphenyltetrazolium Bromide, Thermofisher) stock in water was added into the 96 well-plates to yield a final concentration of 0.5 mg/mL in the culture medium. After 1 h of incubation, the medium that contained the MTT was removed and replaced with dimethyl sulfoxide (DMSO) which dissolved the blue formazan product of the MTT with 30 min more incubation. The resulting solution was measured by using a microplate reader (BMG Polarstar Optima) at a wavelength of 570 nm. The optical density (OD) values were recorded and cell viability was calculated based on the OD readings normalized to the untreated cells.

### In vivo effects of BING on medaka fish

The efficacy of BING to protect medaka against infection was characterized using host resistance assays with intraperitoneal (i.p.) injection of bacteria using a micro-injection machine (PV820 Pneumatic PicoPump)^73^. The fish were anesthetized in MS222 for 30 s before microinjection. In one experiment, 80 medaka fish (male, 6 months) were used and divided randomly into four groups (20 fish in each group): Group 1 was injected with 1 µL of PBS; Group 2 was injected with 1 µL of BING (1 mg/mL), Group 3 was injected with 1 µL of bacteria (5 × 10^7^ CFU/mL); and Group 4 was injected with bacteria and BING. In another experiment, 60 fish were used and divided into three groups: injected with 1 µL of PBS, ampicillin (2.1 mg/mL) or BING (2 mg/mL) respectively. After two hours, 1 µL of bacteria (5 × 10^7^ CFU/mL) was injected into each fish. All of the fishes were kept under a static condition of 26 ± 1 °C, with 14:10 h of a light–dark cycle. The water was replaced every 2 days. The fish were fed three times each day. The number of dead fish was counted on a daily basis until 2 weeks after the injections.

### Effect of temperature and animal serum on the antibacterial activity of BING

To investigate the thermal stability of BING, water-dissolved BING (stock concentration: 80 µg/mL) was incubated at − 20 °C, 4 °C, 25 °C and 90 °C for 30 min, 3 h and 24 h. To compare the stability of BING in the presence of animal serum, BING and CD BING (both at 125 µg/mL) were incubated in 0%, 5% and 10% of fetal bovine serum (Gibco) for 20–22 h at 37 °C. After the incubation, BING was cooled down to room temperature and serially diluted to 6 concentrations. The antimicrobial activity was determined by broth dilution method against *E. coli* (ATCC 10536) as described above.

### Statistical data analysis

Data were displayed as mean ± SD. For the statistical test, either the *t* test (two samples comparison) or ANOVA (multiple group comparisons) was applied in the study. Excel 2007 and GraphPad Prism 6 were used to perform statistical analysis. When two-way ANOVA was applied, Dunnett's multiple comparisons test was performed as well. Log-rank (Mantel-Cox) test was applied for the comparison of survival curves in fish efficacy study. An alpha value *p* < 0.05 (*) or *p* < 0.01 (**) was considered statistically significant.

### Ethic statement

Experiments on the Japanese medaka were carried out in accordance with the directive of the European Union 86/609/EEC and the protocols were approved the Department of Health, HKSRA [(13-11) in DH/HA&P/8/2/5 Pt. 1].

## Supplementary Information

Supplementary Dataset 1.

Supplementary Dataset 2.

Supplementary Information.
