# Supplementary material for: BING, a novel antimicrobial peptide isolated from Japanese medaka plasma, targets bacterial envelope stress response by suppressing cpxR expression
Source: Sci Rep. 2021 Jun 9;11:12219. doi: 10.1038/s41598-021-91765-4 (PMC8190156; doi:10.1038/s41598-021-91765-4)
Supplement: Supplementary file 3 — Supplementary Information. [file 41598_2021_91765_MOESM3_ESM.docx]

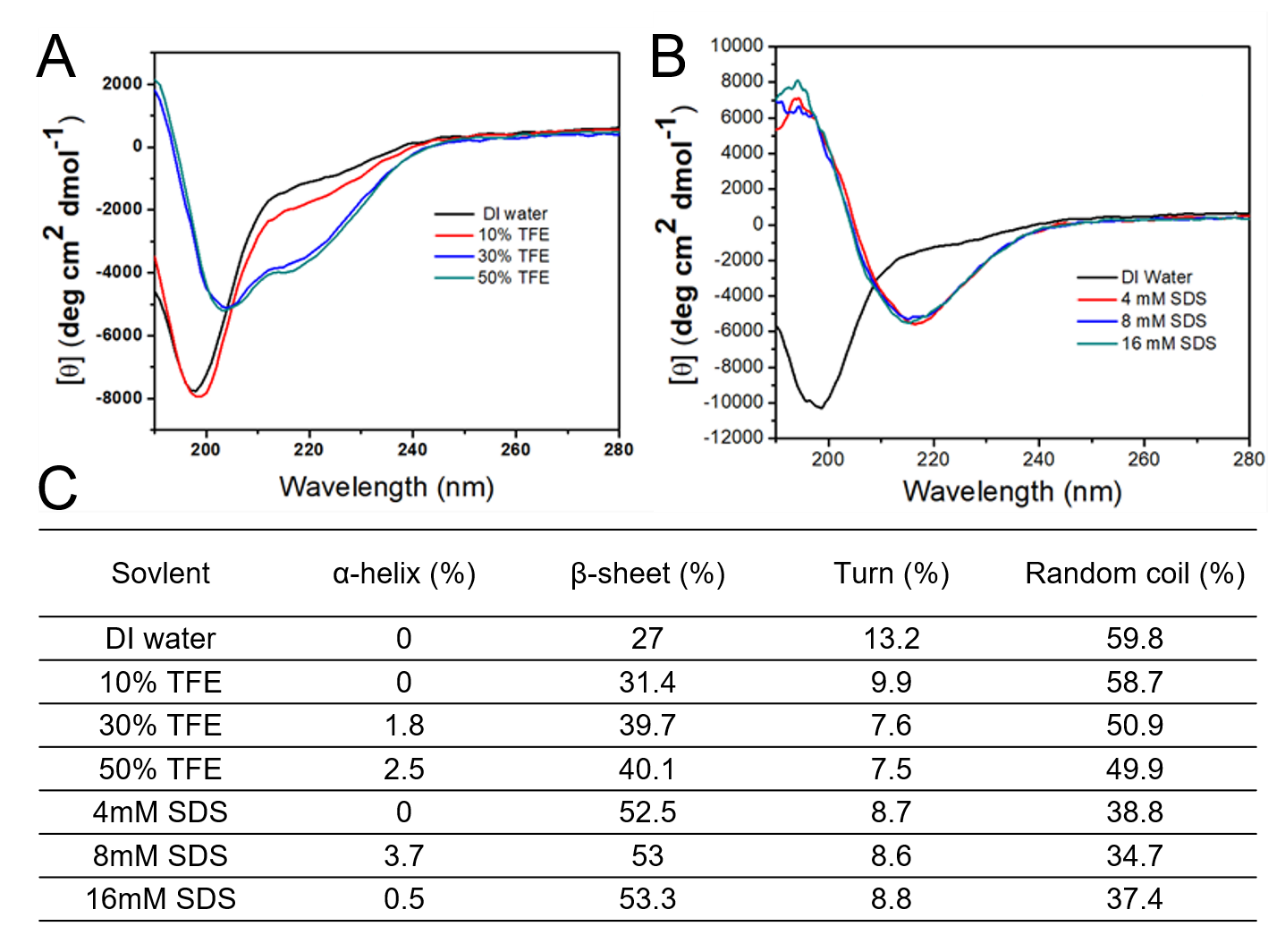


**SUPPLEMENTARY FIGURE 1**: Secondary structure analysis of BING peptide by Far-UV CD spectroscopy. The CD spectra of BING-peptide were measured in aqueous solution with increasing levels of (A) TFE and (B) SDS according to the conditions described in Materials and Methods. (C) The relative contents of secondary structure elements were estimated by CD deconvolution analysis BESTSEL server^1^. [Ɵ], Mean residue ellipticity.


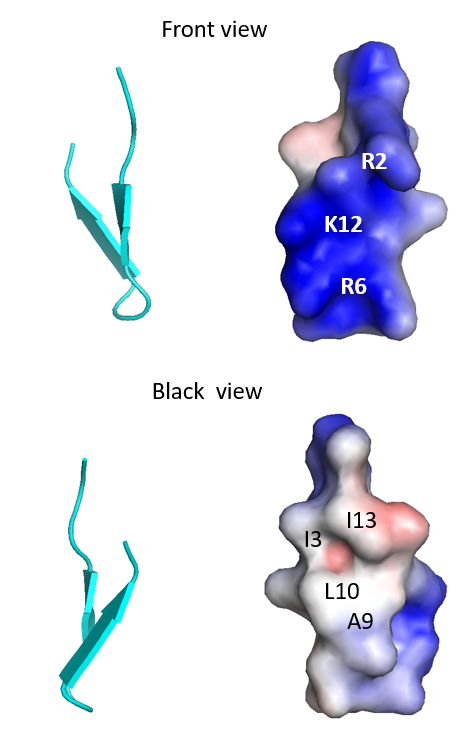


**SUPPLEMENTARY FIGURE 2**: The ribbon diagram of the BING-peptide 3D model (left) and electrostatic potential mapped onto the accessible surface area of the model structure (Right). The 3D model was generated using PEP-FOLD^2^ and visualized by PyMol (DeLano Scientific). The positive electrostatic surface is shown in blue, and the negative surface in shown in red; all surfaces are drawn at ±3 kT/e.


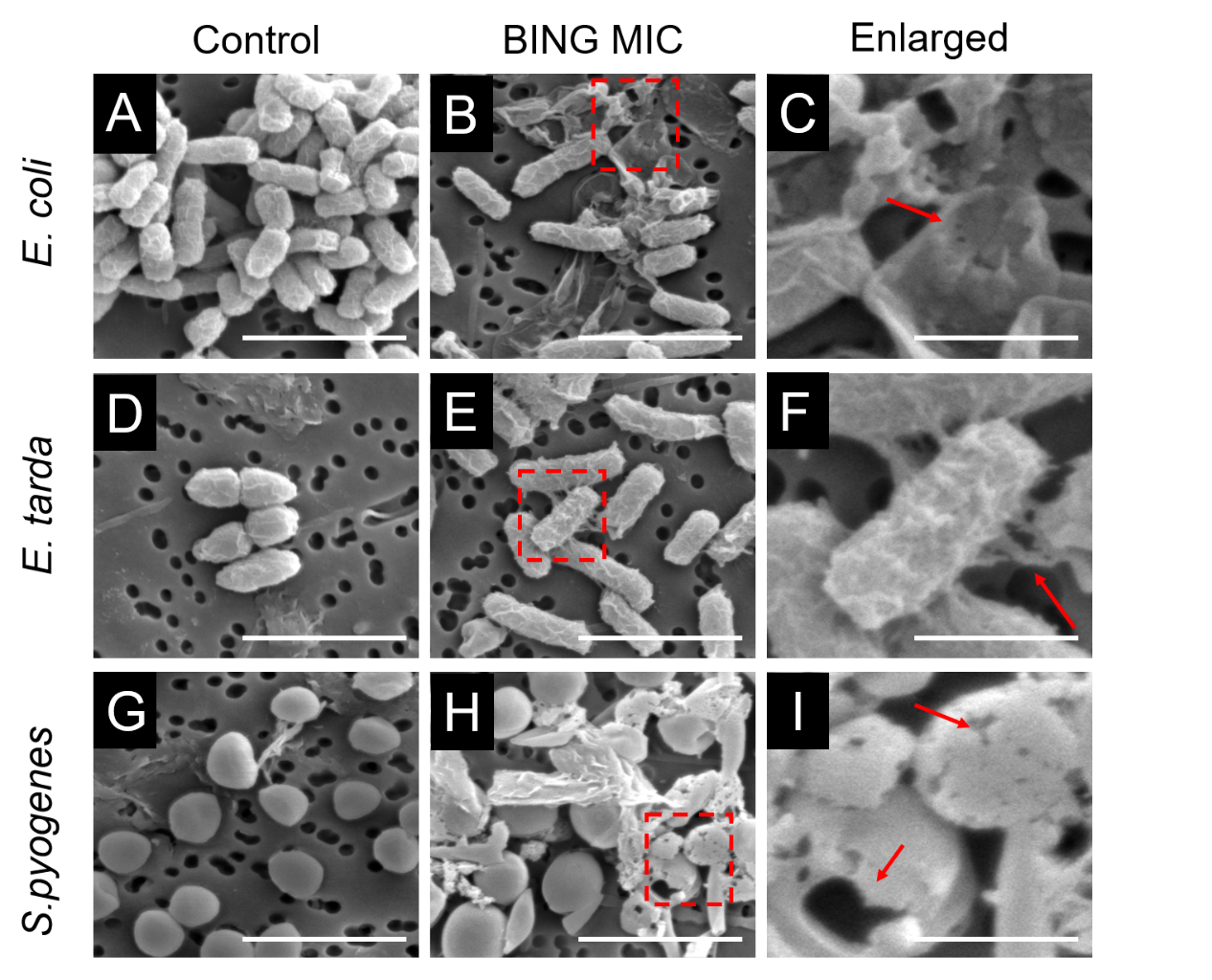


**SUPPLEMENTARY FIGURE 3**: Scanning electron micrographs of *E. coli* (A-C) *E. tarda* (D-F) and *S. pyogenes* (G-I) incubated with BING (7.8 µg/ml for *E. coli*, 10 µg/ml for *E. tarda* and 50 µg/ml for *S. pyogenes*) or culture medium for 16 hours. Bars in panels A, B, D, E, G and H: 3 µm. Bars in panels C, F and I: 1 µm. Representative images from three replicates were shown.


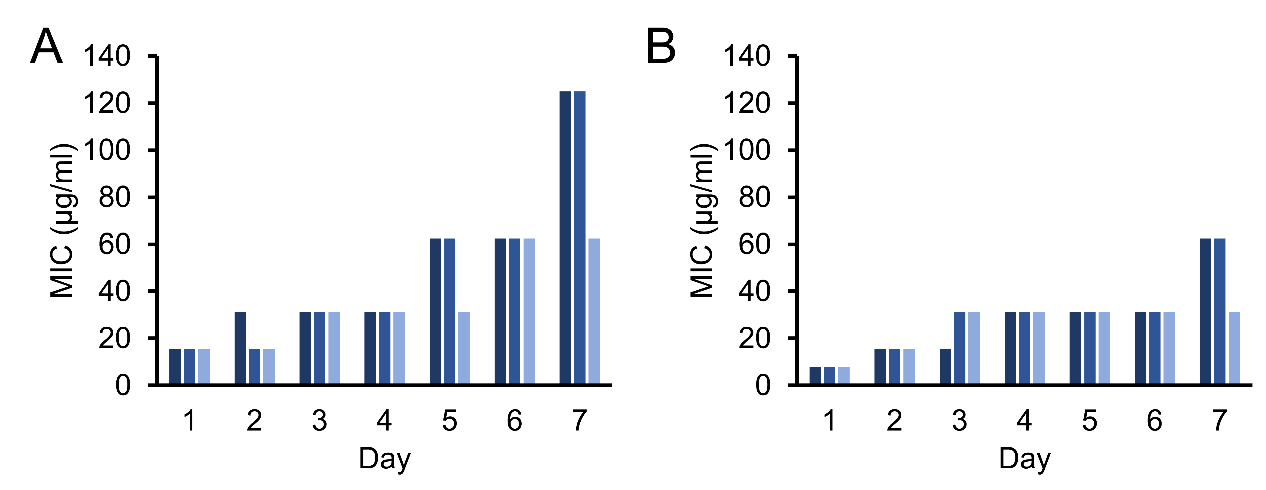


**SUPPLEMENTARY FIGURE 4**: Effect of *cpxR* expression on the development of antibiotic resistance. Each bar represents one of the four replicates in each experiment. Bacteria were cultured in an increasing concentrations of ampicillin and cells survived at the highest antibiotic concentration were passaged daily for 7 days. Data here show the MIC of the selected cells on each day. (A) Widtype *E.coli (*strain 25113). (B) *cpxR* deleted *E. coli* (strain JW3883).

**SUPPLEMETARY TABLE 1: BACTERIAL STRAIN USED IN THIS STUDY**

|  | Bacteria | Strains | Culture media* | Culture temp / ℃ |
| --- | --- | --- | --- | --- |
|  | *Streptococcus faecalis* |  | TSB | 37 |
|  | *Streptococcus pyogenes* | ATCC 14289 | TSB | 37 |
|  | *Staphylococcus aureus* | ATCC 6538 | TSB | 37 |
|  | *Aeromonas hydrophila* | ATCC 49140 | TSB | 28 |
|  | *Vibrio alginolyticus* | ATCC 33840 | TSB | 28 |
|  | *Edwardsiella tarda* | PE 210 | TSB | 28 |
|  | *Enterobacter cloacae* | ATCC BAA-1143 | MHB | 37 |
|  | *Escherichia coli* | ATCC 10536 | NB | 37 |
|  | *Escherichia coli* | BW25113 | NB | 37 |
|  | *E. coli* (pathogenic) |  | NB | 37 |
|  | *Escherichia coli* | JW3883-AM | NB | 37 |
|  | *Acinetobacter baumannii* | ATCC 19606 | MHB | 37 |
|  | *E. coli* | BL21 (DE3) | MHB | 37 |
|  | *Klebsiella pneumoniae* (NDM-1) | ATCC BAA-2470 | MHB | 37 |
|  | *E. coli* (NDM-1) | ATCC BAA-2469 | MHB | 37 |
|  | NDM-1/BL21 (DE3) |  | MHB | 37 |
|  | SHV-1/BL21 (DE3) |  | MHB | 37 |
|  | TEM-1/BL21 (DE3) |  | MHB | 37 |
|  | MCR-1/BL21 (DE3) |  | MHB | 37 |
|  | *Bacillus subtilis* | 168 | MHB | 37 |
|  | *Staphylococcus aureus* | ATCC 29213 | CaMHB | 37 |
|  | Methicillin-resistant *Staphylococcus aureus* | ATCC BAA-41 | CaMHB | 37 |
|  | Multidrug-resistant *Staphylococcus aureus* | ATCC BAA-44 | CaMHB | 37 |
|  | *Staphylococcus epidermidis* | ATCC 12228 | MHB | 37 |
|  | *Pseudomonas aeruginosa* A |  | NB | 37 |

*TSB: Tryptic soy broth; NB: Nutrient broth; MHB: Mueller-Hinton broth; CaMHB: Cation-adjusted Mueller-Hinton broth. NDM-1, SHV-1, TEM-1 or MCR-1 /BL21 are genes cloned in vectors and transformed into *E.coli* BL21 (DE3) strain. E.coli strains BW25113 and JW3883-AM were provided by National Institute of Genetics. These bacteria were provided by Prof. K. Y. Wong (Department of Applied Biology and Chemical Technology and the State Key laboratory of Chirosciences, The Hong Kong Polytechnic University, Hong Kong)*; S. faecalis* and pathogenic *E.coli* were provided by Dr. Richard Y. C. Kong (Department of Biology and Chemistry, City University of Hong Kong)

**SUPPLEMETARY TABLE 2: MAMMALIAN CELL LINE USED IN THIS STUDY**

|  | Cell Line | Source | Organism | Tissue | Cell Type |
| --- | --- | --- | --- | --- | --- |
|  | HeLa | ATCC® CCL-2™ | Human | Cervix | Epithelial |
|  | MCF7 | ATCC® HTB-22™ | Human | Mammary Gland | Epithelial |
|  | MDA-MB | ATCC® CRM-HTB-26™ | Human | Mammary Gland | Epithelial |
|  | H1299 | ATCC® CRL-5803™ | Human | Lung | Epithelial |
|  | MC3T3 E1 | ATCC® CRL-2593™ | Mouse | Calvaria | Preosteoblast |
|  | AG06858 | Coriell cell collection | Human | Foreskin | Fibroblast |

**SUPPLEMETARY TABLE 3:** **PRIMERS USED FOR qRT-PCR**

|  | Primer |  | Sequence (5'-3') |
| --- | --- | --- | --- |
| *E.tarda* | CPXR | Fowrward | AGACGCCGGTCATTATGCTC |
|  |  | Reverse | ACCAGCTCGCGATCATTGAA |
|  | CPXA | Fowrward | TACTGATGCTGCCCAAGCTC |
|  |  | Reverse | GATCACCCGTCCTTCACTGG |
|  | RPOE | Fowrward | CCGGACAGTCCATGATAGCC |
|  |  | Reverse | CGCACCATTGAGTCATTGCC |
|  | 16s rRNA | Fowrward | ACTGAGACACGGTCCAGACTCCTAC |
|  |  | Reverse | TTAACGTTCACACCTTCCTCCCTAC |
| *E.coli* | CPXR | Fowrward | AGGCGCTTGATCTTCTGGAC |
|  |  | Reverse | GGAGATAGTCATCTGCGCCC |
|  | 16s rRNA | Fowrward | GTTAATACCTTTGCTCATTGA |
|  |  | Reverse | ACCAGGGTATCTAATCCTGTT |
| *P. aeruginosa* | 16s rRNA | Fowrward | CAAAACTACTGAGCTAGAGTACG |
|  |  | Reverse | GCCACTGGTGTTCCTTCCTA |
|  | CPXR | Fowrward | TGCTCTCTTTGTACCTGGCG |
|  |  | Reverse | CACCTCGATACCGGAGATGC |
|  | MEXB | Fowrward | GTGTTCGGCTCGCAGTACTC |
|  |  | Reverse | AACCGTCGGGATTGACCTTG |
|  | MEXY | Fowrward | CCGCTACAACGGCTATCCCT |
|  |  | Reverse | AGCGGGATCGACCAGCTTTC |
|  | OPRM | Fowrward | GATCCCCGACTACCAGCGCCCCG |
|  |  | Reverse | ATGCGGTACTGCGCCCGGAAGGC |
| Meakada fish | VPS13D | Fowrward | GGAGGAAATCCCACCCCAAG |
|  |  | Reverse | GGTTGCACTGTTTCGTCCAC |
|  | 18s rRNA | Fowrward | CCTGCGGCTTAATTTGACCC |
|  |  | Reverse | GACAAATCGCTCCACCAACT |

**REFERENCES**

1. Micsonai A, Wien F, Kernya L, *et al.* BeStSel: a web server for accurate protein secondary structure prediction and fold recognition from the circular dichroism spectra. Nucleic Acids Res 2018; 46: W315-W322.

2. Thévenet P, Shen Y, Maupetit J, *et al.* PEP-FOLD: An updated de novo structure prediction server for both linear and disulfide bonded cyclic peptides. Nucleic Acids Res 2012; 40: W288-93.
